# Essential Conditions for the Full Synergy of Probability of Occurrence Distributions

**DOI:** 10.3390/e24070993

**Published:** 2022-07-18

**Authors:** Rubem P. Mondaini, Simão C. de Albuquerque Neto

**Affiliations:** COPPE, Centre of Technology, Federal University of Rio de Janeiro, Rio de Janeiro 21941-901, Brazil; simao.albuquerqueneto@coppe.ufrj.br

**Keywords:** entropy measures, synergy, probabilistic distributions

## Abstract

In this contribution, we specify the conditions for assuring the validity of the synergy of the distribution of probabilities of occurrence. We also study the subsequent restriction on the maximal extension of the strict concavity region on the parameter space of Sharma–Mittal entropy measures, which has been derived in a previous paper in this journal. The present paper is then a necessary complement to that publication. Some applications of the techniques introduced here are applied to protein domain families (Pfam databases, versions 27.0 and 35.0). The results will show evidence of their usefulness for testing the classification work performed with methods of alignment that are used by expert biologists.

## 1. Introduction

We have been working since 2015 on the problem of testing the alignment of protein domain families which are proposed by expert biologists and bioinformaticians. We have found that the use of selected entropy measures is very proficient for testing the results published by those professionals and they favour a rigorous ANOVA statistical analysis [1]. In order to reduce the search space for admissible values of entropy measures, we have emphasized the need for work in the region related to strict concavity of these entropies. This study has been undertaken in a previous work, and we present in Section 2 a summary of those developments. In the present work, we aim to complement the results of a previous publication [2], and a subsequent restriction on the parameter space has to be performed in order to guarantee the synergy of the probability distributions to be tested. Non-synergetic distributions are not worthwhile for working because they will not preserve the fundamental property of getting more information of amino acids into *t*-sets of columns than to sum up the information obtained from individual columns. In Section 3, a brief digression is then made for introducing the Sharma–Mittal class of entropy measures. Section 4 emphasizes the aspects of synergy of the distributions and their consequences for the reduction of the parameter space of Sharma–Mittal entropies. In Section 5, we treat the analysis of the maximal extension of the parameter space, and we repeat the reduction process imposed by the requirement of fully synergetic distributions of Section 4. We conclude the paper in Section 6 by studying the relation of Hölder and generalized Khinchin–Shannon (GKS) inequalities.

## 2. The Construction of the Probabilistic Space

Let us consider a set of $m_{f}$ domains ($m_{f}$ rows) from a chosen family of protein domains. In order to associate a rectangular array with this family, to be taken as its representative in the probabilistic space we are constructing, we specify its number of columns as $n_{f}=n$. This means that among $m_{f}$ rows, we disregard all rows such that the number of their amino acids satisfies $n_{f}<n$ and preserve $m_{f}^{\prime }$ rows whose number of amino acids satisfies $n_{f}\leq n$, but disregard ($n_{f}-n$) amino acids in these $m_{f}^{\prime }$ rows. We then choose *m* rows from among the $m_{f}^{\prime }$ rows to obtain m×n rectangular arrays. There are $m_{f}^{\prime }!/\left[m!\left(m_{f}^{\prime }-m\right)!\right]$ of these m×n rectangular arrays. Any one of them can be used as a representative of the domain family to be analysed in the statistical procedure to be implemented.

The next step is to assign a joint probability of occurrence of a set of variables $a_{1},\ldots ,a_{t}$ in columns $j_{1},\ldots ,j_{t}$ to be given by

(1) $$p_{j_{1}\ldots j_{t}}\left(a_{1},\ldots ,a_{t}\right)=\frac{n_{j_{1}\ldots j_{t}}\left(a_{1},\ldots ,a_{t}\right)}{m}\;,$$

where $n_{j_{1}\ldots j_{t}}\left(a_{1},\ldots ,a_{t}\right)$ stands for the number of occurrences of the set $a_{1},\ldots ,a_{t}$ in the *t* columns of the subarray m×t of the representative array m×n (1≤t≤n). The symbols $a_{1},\ldots ,a_{t}$ will be running over the letters of the one-letter code for the twenty amino acids: $a_{j}\left(1\leq j\leq t\right)\in$ {A, C, D, E, F, G, H, I, K, L, M, N, P, Q, R, S, T, V, W, Y}.

We then have

(2) $$\sum_{a_{1},\ldots ,a_{t}}n_{j_{1}\ldots j_{t}}\left(a_{1},\ldots ,a_{t}\right)\equiv m\;.$$

We also introduce the conditional probabilities of occurrence, which are given implicitly by

(3) $$p_{j_{1}\ldots j_{t}}\left(a_{1},\ldots ,a_{t}\right)\equiv p_{j_{1}\ldots j_{t}}\left(a_{1},\ldots ,a_{t-1}|a_{t}\right)p_{j_{t}}\left(a_{t}\right)\;,$$

where $p_{j_{1}\ldots j_{t}}\left(a_{1},\ldots ,a_{t-1}|a_{t}\right)$ is the probability of occurrence of the amino acids in the columns $j_{1},\ldots ,j_{t-1}$, if the distribution of amino acids in the $j_{t}$-th column is known a priori.

The Bayes’ law for probabilities of occurrence [2,3] can be written as

(4) $$\begin{array}{cc} p_{j_{1}\ldots j_{t}}\left(a_{1},\ldots ,a_{t}\right) & \equiv p_{j_{1}\ldots j_{t}}\left(a_{1},\ldots ,a_{t-1}|a_{t}\right)p_{j_{t}}\left(a_{t}\right)\\ \; & =p_{j_{t}j_{1}\ldots j_{t-1}}\left(a_{t}|a_{1},\ldots ,a_{t-1}\right)p_{j_{1}\ldots j_{t-1}}\left(a_{1},\ldots ,a_{t-1}\right)\\ \; & =p_{j_{t}j_{t-1}j_{1}\ldots j_{t-2}}\left(a_{t},a_{t-1}|a_{1},\ldots ,a_{t-2}\right)p_{j_{1}\ldots j_{t-2}}\left(a_{1},\ldots ,a_{t-2}\right)\\ \; & =\ldots \ldots \ldots \ldots \ldots \ldots \ldots \ldots \ldots \ldots \ldots \ldots \ldots \ldots \ldots \ldots \ldots \ldots \\ \; & =p_{j_{t}\ldots j_{3}j_{1}j_{2}}\left(a_{t},\ldots ,a_{3}|a_{1},a_{2}\right)p_{j_{1}j_{2}}\left(a_{1},a_{2}\right)\\ \; & =p_{j_{t}\cdots j_{2}j_{1}}\left(a_{t},\ldots ,a_{2}|a_{1}\right)p_{j_{1}}\left(a_{1}\right)\\ \; & =p_{j_{t}\ldots j_{1}}\left(a_{t},\ldots ,a_{1}\right)\;. \end{array}$$

The equality of the three first right-side members, as well as the equality of the three last ones, does correspond to the application of Bayes’ law [2,3]. The symmetries for the joint probability distribution $p_{j_{1}\ldots j_{t}}\left(a_{1},\ldots ,a_{t}\right)$ are due to the ordering of the columns for the distributions of amino acids.

From the ordering $j_{1}<j_{2}<\ldots <j_{t}$, the values assumed by the variables $j_{1},\ldots ,j_{t}$ are respectively given by

(5) $$\begin{array}{cc} j_{1} & =1,2,\ldots ,n-t+1\\ j_{2} & =j_{1}+1,j_{1}+2,\ldots ,n-t+2\\ \; & \;\vdots \;,\;\;1\leq t\leq n\\ j_{t-1} & =j_{t-2}+1,j_{t-2}+2,\ldots ,n-1\\ j_{t} & =j_{t-1}+1,j_{t-1}+2,\ldots ,n\;. \end{array}$$

We then have nt=n!t!(n−t)! geometric objects $p_{j_{1}\ldots j_{t}}\left(a_{1},\ldots ,a_{t}\right)$ of *t* columns and ${\left(20\right)}^{t}$ components each.

## 3. The Sharma–Mittal Class of Entropy Measures

As emphasized in Ref. [2], the introduction of random variable functions such as entropy measures associated with the probabilities of occurrence, is suitable to provide an analysis of the evolution of these probabilities through the regions of the parameter space of entropies. The class of Sharma–Mittal entropy measures seems to be particularly adapted to this task when related to the occurrence of amino acids in the objects $p_{j_{1}\ldots j_{t}}\left(a_{1},\ldots ,a_{t}\right)$. The thermodynamic interpretation of the notion of entropy greatly helps to classify the distribution of its values associated with protein domain databases and to interpret its evolution through the Fokker–Planck equations to be treated in forthcoming articles in this line of research.

The two-parameter Sharma–Mittal class of entropy measures is usually given by

(6) $${\left(SM\right)}_{j_{1}\ldots j_{t}}^{\left(r,s\right)}=\frac{{\left(\alpha_{j_{1}\ldots j_{t}}^{\left(s\right)}\right)}^{\frac{1-r}{1-s}}-1}{1-r}\;;\;{\left(SM\right)}_{j_{t}}^{\left(r,s\right)}=\frac{{\left(\alpha_{j_{t}}^{\left(s\right)}\right)}^{\frac{1-r}{1-s}}-1}{1-r}\;,$$

where

(7) $$\alpha_{j_{1}\ldots j_{t}}^{\left(s\right)}=\sum_{a_{1},\ldots ,a_{t}}{\left[p_{j_{1}\ldots j_{t}}\left(a_{1},\ldots ,a_{t}\right)\right]}^{s}\;;\;\alpha_{j_{t}}^{\left(s\right)}=\sum_{a_{t}}{\left[p_{j_{t}}\left(a_{t}\right)\right]}^{s}\;.$$

The parameters *r*, *s* must bound a region corresponding to a strict concavity in the parameter space. A necessary requirement to be satisfied [3] is

(8) $$\frac{\partial^{2}{\left(SM\right)}_{j_{1}\ldots j_{t}}^{\left(r,s\right)}}{\partial {\left(p_{j_{1}\ldots j_{t}}\left(a_{1},\ldots ,a_{t}\right)\right)}^{2}}=s{\left(\alpha_{j_{1}\ldots j_{t}}^{\left(s\right)}\right)}^{\frac{s-r}{1-s}}\cdot {\left[p_{j_{1}\ldots j_{t}}\left(a_{1},\ldots ,a_{t}\right)\right]}^{s-2}\cdot \left[\frac{s(s-r)}{{\left(1-s\right)}^{2}}\;{\hat{p}}_{j_{1}\ldots j_{t}}\left(a_{1},\ldots ,a_{t}\right)-1\right]<0\;,$$

where ${\hat{p}}_{j_{1}\ldots j_{t}}\left(a_{1},\ldots ,a_{t}\right)$ stands for the escort probability associated with the joint probability $p_{j_{1}\ldots j_{t}}\left(a_{1},\ldots ,a_{t}\right)$, or,

(9) $${\hat{p}}_{j_{1}\ldots j_{t}}\left(a_{1},\ldots ,a_{t}\right)=\frac{{\left[p_{j_{1}\ldots j_{t}}\left(a_{1},\ldots ,a_{t}\right)\right]}^{s}}{\alpha_{j_{1}\ldots j_{t}}^{\left(s\right)}}\;;\;{\hat{p}}_{j_{t}}\left(a_{t}\right)=\frac{{\left[p_{j_{t}}\left(a_{t}\right)\right]}^{s}}{\alpha_{j_{t}}^{\left(s\right)}}\;.$$

Equation (8) leads to

(10) r≥s>0.

Some special cases of one-parameter entropies are commonplace in the scientific literature [3,4,5,6,7,8,9]:

The r=s region is the domain of the Havrda–Charvat [6] entropy measure $H_{j_{1}\ldots j_{t}}^{\left(s\right)}$,

(11) $$H_{j_{1}\ldots j_{t}}^{\left(s\right)}=\frac{\alpha_{j_{1}\ldots j_{t}}^{\left(s\right)}-1}{1-s}\;.$$

The r=2−s, 0≤s≤1, region will stand for the domain of the Landsberg–Vedral [7] entropy measure, $L_{j_{1}\ldots j_{t}}^{\left(s\right)}$,

(12) $$L_{j_{1}\ldots j_{t}}^{\left(s\right)}=\frac{\alpha_{j_{1}\ldots j_{t}}^{\left(s\right)}-1}{\left(1-s\right)\alpha_{j_{1}\ldots j_{t}}^{\left(s\right)}}\equiv \frac{H_{j_{1}\ldots j_{t}}^{\left(s\right)}}{\alpha_{j_{1}\ldots j_{t}}^{\left(s\right)}}\;.$$

The Renyi $R_{j_{1}\ldots j_{t}}^{\left(s\right)}$ [8] and the “non-extensive” Gaussian [9] $G_{j_{1}\ldots j_{t}}^{\left(r\right)}$ entropy measures are obtained from limit processes:

(13) $$\begin{array}{cc} R_{j_{1}\ldots j_{t}}^{\left(s\right)} & \equiv \lim_{r\to 1}\frac{{\left(\alpha_{j_{1}\ldots j_{t}}^{\left(s\right)}\right)}^{\frac{1-r}{1-s}}-1}{1-r}=\lim_{r\to 1}\frac{\frac{d}{dr}\left[{\left(\alpha_{j_{1}\ldots j_{t}}^{\left(s\right)}\right)}^{\frac{1-r}{1-s}}-1\right]}{\frac{d}{dr}\left(1-r\right)}=\lim_{r\to 1}\frac{e^{\frac{1-r}{1-s}\cdot \log\alpha_{j_{1}\ldots j_{t}}^{\left(s\right)}}\cdot \left(\frac{-1}{1-s}\right)\log\alpha_{j_{1}\ldots j_{t}}^{s}}{-1}\\ \; & =\frac{\log\alpha_{j_{1}\ldots j_{t}}^{\left(s\right)}}{1-s}\;. \end{array}$$

$$\begin{array}{cc} G_{j_{1}\ldots j_{t}}^{\left(r\right)} & \equiv \lim_{s\to 1}\frac{{\left(\alpha_{j_{1}\ldots j_{t}}^{\left(s\right)}\right)}^{\frac{1-r}{1-s}}-1}{1-r}=\frac{1}{1-r}\left[\exp\left(\left(1-r\right)\cdot \lim_{s\to 1}\frac{\frac{d}{ds}\log\alpha_{j_{1}\ldots j_{t}}^{\left(s\right)}}{\frac{d}{ds}\left(1-s\right)}\right)-1\right]\\ \; & =\frac{1}{1-r}\left[\exp\left(-\left(1-r\right)\cdot \lim_{s\to 1}\frac{\frac{d}{ds}\alpha_{j_{1}\ldots j_{t}}^{\left(s\right)}}{\alpha_{j_{1}\ldots j_{t}}^{\left(s\right)}}\right)-1\right]\;. \end{array}$$

After using the definition of $\alpha_{j_{1}\ldots j_{t}}^{\left(s\right)}$, Equation (7), and $\lim_{s\to 1}\alpha_{j_{1}\ldots j_{t}}^{\left(s\right)}=1$ from Equations (1) and (2), we get:

(14) $$G_{j_{1}\ldots j_{t}}^{\left(r\right)}=\frac{e^{\left(1-r\right)S_{j_{1}\ldots j_{t}}}-1}{1-r}\;,$$

where $S_{j_{1}\ldots j_{t}}$ is the Gibbs–Shannon entropy measure

(15) $$S_{j_{1}\ldots j_{t}}=-\;\;\;\sum_{a_{1},\ldots ,a_{t}}p_{j_{1}\ldots j_{t}}\left(a_{1},\ldots ,a_{t}\right)\;\log p_{j_{1}\ldots j_{t}}\left(a_{1},\ldots ,a_{t}\right)\;.$$

The Gibbs–Shannon entropy measure, Equation (15), is also obtained by taking the convenient limits of the special cases of Sharma–Mittal entropies, Equations (11)–(14):

(16) $$\lim_{s\to 1}H_{j_{1}\ldots j_{t}}^{\left(s\right)}=\lim_{s\to 1}L_{j_{1}\ldots j_{t}}^{\left(s\right)}=\lim_{s\to 1}R_{j_{1}\ldots j_{t}}^{\left(s\right)}=\lim_{r\to 1}G_{j_{1}\ldots j_{t}}^{\left(r\right)}=S_{j_{1}\ldots j_{t}}\;.$$

We shall analyse in the next section the structure of the two-parameter space of Sharma–Mittal entropy by taking into consideration these special cases.

We are now reminded that for the limit of Gibbs–Shannon entropy, a conditional entropy measure is defined [3] by

(17) $$S_{j_{1}\ldots j_{t-1}|j_{t}}=-\;\;\;\sum_{a_{1},\ldots ,a_{t}}p_{j_{t}}\left(a_{t}\right)p_{j_{1}\ldots j_{t}}\left(a_{1},\ldots ,a_{t-1}|a_{t}\right)\;\log p_{j_{1}\ldots j_{t}}\left(a_{1},\ldots ,a_{t-1}|a_{t}\right)\;.$$

We then have analogously for the conditional Sharma–Mittal entropy measure [3]

(18) $${\left(SM\right)}_{j_{1}\ldots j_{t-1}|j_{t}}=\frac{{\left(\sum_{a_{1},\ldots ,a_{t}}{\hat{p}}_{j_{t}}\left(a_{t}\right){\left[p_{j_{1}\ldots j_{t}}\left(a_{1},\ldots ,a_{t-1}|a_{t}\right)\right]}^{s}\right)}^{\frac{1-r}{1-s}}-1}{1-r}\;.$$

It is easy to show by trivial calculation that, analogously to Equation (16), we will have

(19) $$\lim_{s\to 1}\;\lim_{r\to s}\;{\left(SM\right)}_{j_{1}\ldots j_{t-1}|j_{t}}=\lim_{s\to 1}\lim_{r\to 2-s}{\left(SM\right)}_{j_{1}\ldots j_{t-1}|j_{t}}=\lim_{s\to 1}\;\lim_{r\to 1}\;{\left(SM\right)}_{j_{1}\ldots j_{t-1}|j_{t}}=\lim_{r\to 1}\;\lim_{s\to 1}\;{\left(SM\right)}_{j_{1}\ldots j_{t-1}|j_{t}}=S_{j_{1}\ldots j_{t-1}|j_{t}}\;.$$

From Equations (6), (7) and (18) and the application of the Bayes’ law, Equation (4), we can write

(20) $${\left(SM\right)}_{j_{1}\ldots j_{t}}={\left(SM\right)}_{j_{t}}+{\left(SM\right)}_{j_{1}\ldots j_{t-1}|j_{t}}+\left(1-r\right){\left(SM\right)}_{j_{t}}{\left(SM\right)}_{j_{1}\ldots j_{t-1}|j_{t}}\;.$$

## 4. Aspects of Synergy and the Reduction of the Parameter Space for Fully Synergetic Distributions

For the Gibbs–Shannon entropy measure, the inequality written by A. Y. Khinchin [3,10] is

(21) $$S_{j_{1}\ldots j_{t-1}|j_{t}}\leq S_{j_{1}\ldots j_{t-1}}\;.$$

This inequality would be described by Khinchin as: “On the average, the knowledge a priori of the distribution on the column $j_{t}$ can only decrease the uncertainty of the distribution on the $j_{1},\ldots ,j_{t-1}$ columns”. We can write an analogous inequality for the Sharma–Mittal class of entropies

(22) $${\left(SM\right)}_{j_{1}\ldots j_{t-1}|j_{t}}\leq {\left(SM\right)}_{j_{1}\ldots j_{t-1}}\;.$$

We then get from Equations (20) and (22)

(23) $${\left(SM\right)}_{j_{1}\ldots j_{t}}\leq {\left(SM\right)}_{j_{t}}+{\left(SM\right)}_{j_{1}\ldots j_{t-1}}+\left(1-r\right){\left(SM\right)}_{j_{t}}{\left(SM\right)}_{j_{1}\ldots j_{t-1}}\;.$$

After iteration of this equation, t→t−1→t−2→…, we can also write

(24) $${\left(SM\right)}_{j_{1}\ldots j_{t}}\leq \frac{\prod_{l=1}^{t}\left[1+\left(1-r\right){\left(SM\right)}_{j_{l}}\right]-1}{1-r}\;.$$

The inequalities in (21)–(24) are associated with what are called “synergetic conditions”. In this section, we also derive the fully synergetic conditions as GKS inequalities.

After using Equations (7) and (9) in Equation (23), we get

(25) $$\frac{{\left(\alpha_{j_{1}\ldots j_{t}}^{\left(s\right)}\right)}^{\frac{1-r}{1-s}}}{1-r}\leq \frac{{\left(\alpha_{j_{t}}^{\left(s\right)}\right)}^{\frac{1-r}{1-s}}\cdot {\left(\alpha_{j_{1}\ldots j_{t-1}}^{\left(s\right)}\right)}^{\frac{1-r}{1-s}}}{1-r}\;,$$

and after iteration and use of Equation (24)

(26) $$\frac{{\left(\alpha_{j_{1}\ldots j_{t}}^{\left(s\right)}\right)}^{\frac{1-r}{1-s}}}{1-r}\leq \frac{\prod_{l=1}^{t}{\left(\alpha_{j_{l}}^{\left(s\right)}\right)}^{\frac{1-r}{1-s}}}{1-r}\;.$$

**Figure 1:**
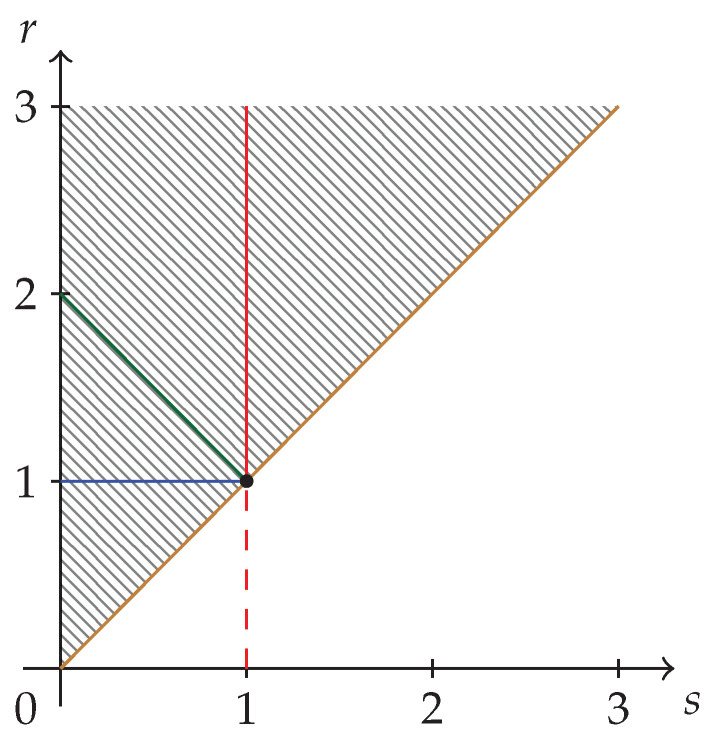
The strict concavity region C={(s,r)|r≥s>0} of Sharma–Mittal class of entropy measures. It is the epigraph of the curve (r=s), and this corresponds to the Havrda–Charvat entropy, which is depicted in brown. The Landsberg–Vedral’s (r=2−s), Renyi’s (r=1), and “non-extensive” Gaussian’s (s=1) are depicted in green, blue, and red, respectively.

The hatched region of strict concavity in the parameter space of Sharma–Mittal entropies, C={(s,r)|r≥s>0}, is depicted in Figure 1. The special cases corresponding to Havrda–Charvat’s (r=s), Landsberg–Vedral’s (r=2−s), Renyi’s (r=1), and “non-extensive” Gaussian’s (s=1) entropies are also represented.

We can identify three subregions in Figure 1. They will correspond to

(27) $$R_{I}=\left\{\left(s,r\right)\;|\;1>r\geq s>0\right\}\;\;\Rightarrow \;\;\alpha_{j_{1}\ldots j_{t}}^{\left(s<1\right)}\leq \prod_{l=1}^{t}\alpha_{j_{t}}^{\left(s<1\right)}\;,$$

(28) $$R_{\mathrm{II}}=\left\{\left(s,r\right)\;|\;r\geq s>1\right\}\;\;\Rightarrow \;\;\alpha_{j_{1}\ldots j_{t}}^{\left(s>1\right)}\geq \prod_{l=1}^{t}\alpha_{j_{t}}^{\left(s>1\right)}\;,$$

(29) $$R_{\mathrm{III}}=\left\{\left(s,r\right)\;|\;r>1>s>0\right\}\;\;\Rightarrow \;\;\alpha_{j_{1}\ldots j_{t}}^{\left(s<1\right)}\leq \prod_{l=1}^{t}\alpha_{j_{t}}^{\left(s<1\right)}\;$$

where the ordering of α-symbols has been obtained from Equation (26). The subregions R${}_{I}$ and R${}_{\mathrm{III}}$ are what we call fully synergetic subregions, and the corresponding inequalities are the GKS inequalities [2].

**Figure 2:**
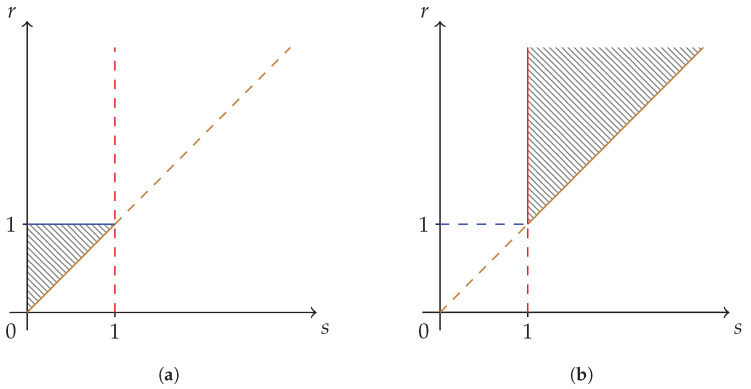

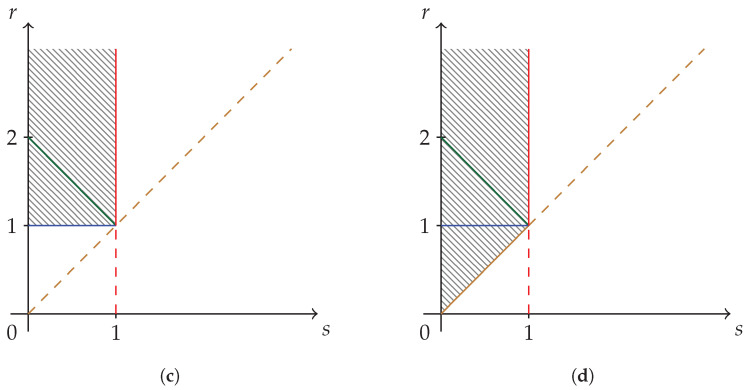
Subregions of the strict concavity region C={(s,r)|r≥s>0} of the Sharma–Mittal class of entropy measures. (**a**) Khinchin–Shannon subregion R${}_{I}$—fully synergetic.; (**b**) The non-synergetic subregion R${}_{\mathrm{II}}$; (**c**) Khinchin–Shannon subregion R${}_{\mathrm{III}}$—fully synergetic; (**d**) Khinchin–Shannon subregion $R_{I}\cup R_{\mathrm{III}}$—fully synergetic. The reduced subregion $R_{I}\cup R_{\mathrm{III}}$ of Figure 1 is obtained by taking into consideration fully synergetic distributions only.

The subregions R${}_{I}$, R${}_{\mathrm{II}}$, and R${}_{\mathrm{III}}$ are depicted in Figure 2a–c, respectively. The union of subregions R${}_{I}$ and R${}_{\mathrm{III}}$ is the fully synergetic Khinchin–Shannon restriction to be imposed on the strict concavity region of Figure 1 and it is depicted in Figure 2d below.

## 5. The Maximal Extension of the Parameter Space and Its Reduction for Fully Synergetic Distribution

In Figure 1 and Figure 2d, we have depicted the structure of the strict concavity region for Sharma–Mittal entropy measures and its reduction to a subregion by the application of the requirement of fully synergetic distributions, respectively. Our analysis has used a coarse-grained approach to concavity given by Equations (8) and (10). We now introduce some necessary refinements for characterizing the probability of occurrence in subarrays of *m* rows and *t* columns, m×t. For *t* columns, there are ${\left(20\right)}^{t}$ possibilities of occurrence of amino acids, which could be a large number, but we could count not individual amino acids, but groups of *t*-sets of amino acids (μ-groups) which appear on the *m* rows of the m×t array. We characterize these μ-groups by μ=1,…,m, from all equal μ-groups (μ=1) to *m* different μ-groups (μ=m). We also call $q_{\mu }$, the number of equal *t*-sets of a given μ-group.

In Equation (2), the sum is over all the amino acids that make up the geometric object defined in Equation (1), the probability of occurrence. We can now perform the sum over μ-groups and write

(30) $$\sum_{a_{1}^{q_{\mu }},\ldots ,a_{t}^{q_{\mu }}}p_{j_{1}\ldots j_{t}}\left(a_{1}^{q_{\mu }},\ldots ,a_{t}^{q_{\mu }}\right)=\sum_{a_{1}^{q_{\mu }},\ldots ,a_{t}^{q_{\mu }}}\frac{n_{j_{1}\ldots j_{t}}\left(a_{1}^{q_{\mu }},\ldots ,a_{t}^{q_{\mu }}\right)}{m}=\sum_{\mu =1}^{m}\frac{q_{\mu }}{m}=1\;,$$

where $\left(a_{1}^{q_{\mu }},\ldots ,a_{t}^{q_{\mu }}\right)$ are the *t*-sets of a μ-group. We also have from Equation (7)

(31) $$\sum_{a_{1}^{q_{\mu }},\ldots ,a_{t}^{q_{\mu }}}{\left[p_{j_{1}\ldots j_{t}}\left(a_{1}^{q_{\mu }},\ldots ,a_{t}^{q_{\mu }}\right)\right]}^{s}=\sum_{a_{1}^{q_{\mu }},\ldots ,a_{t}^{q_{\mu }}}{\left[\frac{n_{j_{1}\ldots j_{t}}\left(a_{1}^{q_{\mu }},\ldots ,a_{t}^{q_{\mu }}\right)}{m}\right]}^{s}=\sum_{\mu =1}^{m}{\left(\frac{q_{\mu }}{m}\right)}^{s}=\alpha_{j_{1}\ldots j_{t}}^{\left(s\right)}\;.$$

From Equations (30) and (31), we can now proceed to the calculation of the Hessian matrix for Sharma–Mittal entropy measures. We have for the first derivative of ${\left(SM\right)}_{j_{1}\ldots j_{t}}$

(32) $$\frac{\partial {\left(SM\right)}_{j_{1}\ldots j_{t}}}{\partial p_{j_{1}\ldots j_{t}}\left(a_{1}^{q_{\mu }},\ldots ,a_{t}^{q_{\mu }}\right)}=\frac{s}{1-s}{\left(\alpha_{j_{1}\ldots j_{t}}^{\left(s\right)}\right)}^{\frac{s-r}{1-s}}{\left[p_{j_{1}\ldots j_{t}}\left(a_{1}^{q_{\mu }},\ldots ,a_{t}^{q_{\mu }}\right)\right]}^{s-1}\;.$$

We then have for a generic element of the Hessian matrix [2]

(33) $$\begin{array}{cc} H_{q_{\mu }q_{\nu }} & =\frac{\partial^{2}{\left(SM\right)}_{j_{1}\ldots j_{t}}}{\partial p_{j_{1}\ldots j_{t}}\left(a_{1}^{q_{\mu }},\ldots ,a_{t}^{q_{\mu }}\right)\partial p_{j_{1}\ldots j_{t}}\left(a_{1}^{q_{\nu }},\ldots ,a_{t}^{q_{\nu }}\right)}\\ \; & =s{\left(\alpha_{j_{1}\ldots j_{t}}^{\left(s\right)}\right)}^{\frac{s-r}{1-s}}{\left[p_{j_{1}\ldots j_{t}}\left(a_{1}^{q_{\mu }},\ldots ,a_{t}^{q_{\mu }}\right)\right]}^{s-2}\left[\frac{s(s-r)}{{\left(1-s\right)}^{2}}\frac{p_{j_{1}\ldots j_{t}}\left(a_{1}^{q_{\mu }},\ldots ,a_{t}^{q_{\mu }}\right)}{p_{j_{1}\ldots j_{t}}\left(a_{1}^{q_{\nu }},\ldots ,a_{t}^{q_{\nu }}\right)}\cdot {\hat{p}}_{j_{1}\ldots j_{t}}\left(a_{1}^{q_{\nu }},\ldots ,a_{t}^{q_{\nu }}\right)-\delta_{\mu \nu }\right]\;, \end{array}$$

where ${\hat{p}}_{j_{1}\ldots j_{t}}\left(a_{1}^{q_{\mu }},\ldots ,a_{t}^{q_{\mu }}\right)$ is the escort probability associated to $p_{j_{1}\ldots j_{t}}\left(a_{1}^{q_{\mu }},\ldots ,a_{t}^{q_{\mu }}\right)$, or

(34) $${\hat{p}}_{j_{1}\ldots j_{t}}\left(a_{1}^{q_{\mu }},\ldots ,a_{t}^{q_{\mu }}\right)\equiv \frac{{\left[p_{j_{1}\ldots j_{t}}\left(a_{1}^{q_{\mu }},\ldots ,a_{t}^{q_{\mu }}\right)\right]}^{s}}{\sum_{b_{1}^{q_{\nu }},\ldots ,b_{t}^{q_{\nu }}}p_{j_{1}\ldots j_{t}}\left(b_{1}^{q_{\nu }},\ldots ,b_{t}^{q_{\nu }}\right)}\;.$$

The principal minors are given by

(35) $$\begin{array}{cc} \det H_{q_{\mu }q_{\nu }}\left(\mu ,\nu =1,\ldots ,k\right)={\left(-1\right)}^{k-1}s^{k}{\left(\alpha_{j_{1}\ldots j_{t}}^{\left(s\right)}\right)}^{\frac{k(s-r)}{1-s}}{\left[\prod_{\mu =1}^{k}p_{j_{1}\ldots j_{t}}\left(a_{1}^{q_{\mu }},\ldots ,a_{t}^{q_{\mu }}\right)\right]}^{s-2} & \\ \cdot \left[\frac{s(s-r)}{{\left(1-s\right)}^{2}}\sum_{\mu =1}^{k}{\hat{p}}_{j_{1}\ldots j_{t}}\left(a_{1}^{q_{\mu }},\ldots ,a_{t}^{q_{\mu }}\right)-1\right]\;,\;k=1,\ldots ,m\;, & \end{array}$$

and we have

(36) $$\sum_{\mu =1}^{k}{\hat{p}}_{j_{1}\ldots j_{t}}\left(a_{1}^{q_{\mu }},\ldots ,a_{t}^{q_{\mu }}\right)=\sum_{\mu =1}^{k}\frac{{\left[p_{j_{1}\ldots j_{t}}\left(a_{1}^{q_{\mu }},\ldots ,a_{t}^{q_{\mu }}\right)\right]}^{s}}{\sum_{\mu =1}^{m}{\left(\frac{q_{\mu }}{m}\right)}^{s}}=\frac{\sum_{\mu =1}^{k}{\left(\frac{q_{\mu }}{m}\right)}^{s}}{\sum_{\mu =1}^{m}{\left(\frac{q_{\mu }}{m}\right)}^{s}}\equiv \sigma_{k}\left(s\right)$$

according to Equation (31).

From Equations (35) and (36), the requirement of strict concavity will lead to

(37) $$\frac{s(s-r)}{{\left(1-s\right)}^{2}}\;\sigma_{k}\left(s\right)-1<0\;.$$

We then have

(38) $$\det H_{q_{\mu }q_{\nu }}\left(\mu ,\nu =1,\ldots ,k\right)<0\;,\;k\;\mathrm{odd};\;>0\;,\;k\;\mathrm{even}.$$

This does correspond to the criterion of negative definiteness of the Hessian matrix for strict concavity of multivariate functions [11].

Each *k*-value is associated with the *k*-epigraph region, which is the *k*-extension of the strict concavity region presented in Figure 1. These regions are given by

(39) $$C_{k_{\max}}=\left\{\left(s,r\right)\;|\;r\geq s>0\right\}\cup \left\{\left(s,r\right)\;|\;s>r>s-\frac{{\left(1-s\right)}^{2}}{s\;\sigma_{k}\left(s\right)}\right\}\;,\;\;\;k=1,\ldots ,m\;.$$

The greatest lower bound of the sequence of *k*-curves is given by $\sigma_{m}\left(s\right)=1$. We then have

(40) $$r_{m}\left(s\right)=2-\frac{1}{s}\;.$$

We can then write for the maximal extended region of strict concavity

(41) $$C_{\max}=\left\{\left(s,r\right)\;|\;r\geq s>0\right\}\cup \left\{\left(s,r\right)\;|\;s>r>2-\frac{1}{s}\right\}\;.$$

**Figure 3:**
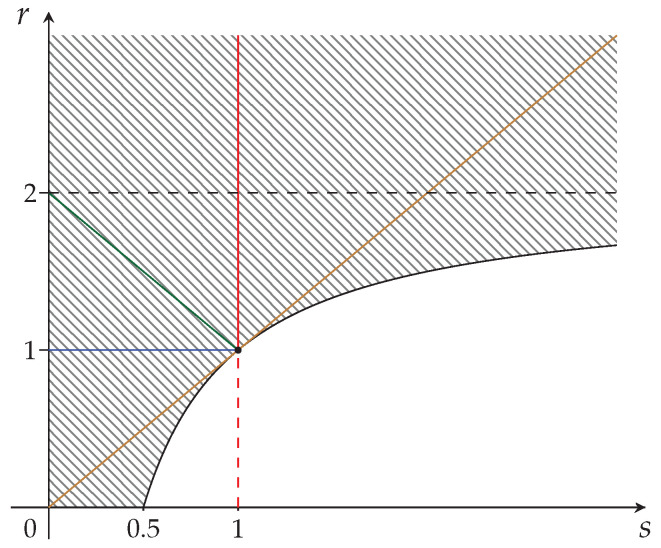
The maximal strict concavity region of the Sharma–Mittal class of entropy measures. The hatched region is the epigraph of the curve r=2−1/s which is depicted in black. The Havrda–Charvat (r=s) region is in brown. The Landsberg–Vedral (r=2−s), Renyi (r=1), and “non-extensive” Gaussian (s=1) regions are depicted in green, blue, and red, respectively.

The region corresponding to Equation (41) is depicted in Figure 3 below.

We are now ready to undertake the application of restrictions for fully synergetic distributions (validity of GKS inequalities) to the maximal strict concavity region of Figure 3.

We start by identifying two regions included in Figure 3. They will be given by

(42) $$R_{\mathrm{IV}}=\left\{\left(s,r\right)\;|\;1>s>r\geq 2-\frac{1}{s}>0\right\}\;\;\Rightarrow \;\;\alpha_{j_{1}\ldots j_{t}}^{\left(s<1\right)}\leq \prod_{l=1}^{t}\alpha_{j_{t}}^{\left(s<1\right)}\;,$$

(43) $$R_{V}=\left\{\left(s,r\right)\;|\;s>r\geq 2-\frac{1}{s}>1\right\}\;\;\Rightarrow \;\;\alpha_{j_{1}\ldots j_{t}}^{\left(s>1\right)}\geq \prod_{l=1}^{t}\alpha_{j_{t}}^{\left(s>1\right)}\;.$$

**Figure 4:**
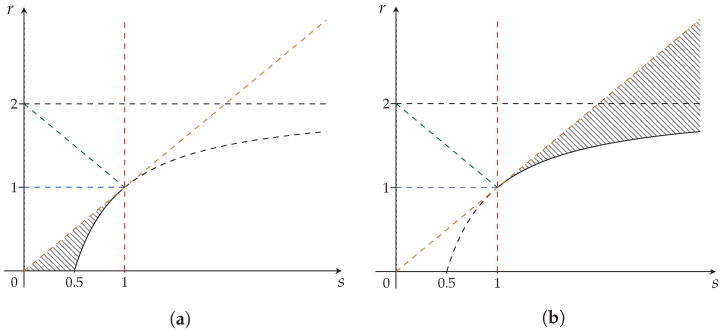
Subregions of the maximal strict concavity region of the Sharma–Mittal class of entropy measures (Figure 3). (**a**) Khinchin–Shannon subregion R${}_{\mathrm{IV}}$—fully synergetic; (**b**) The non-synergetic subregion R${}_{V}$.

These regions are depicted in Figure 4a,b, respectively.

**Figure 5:**
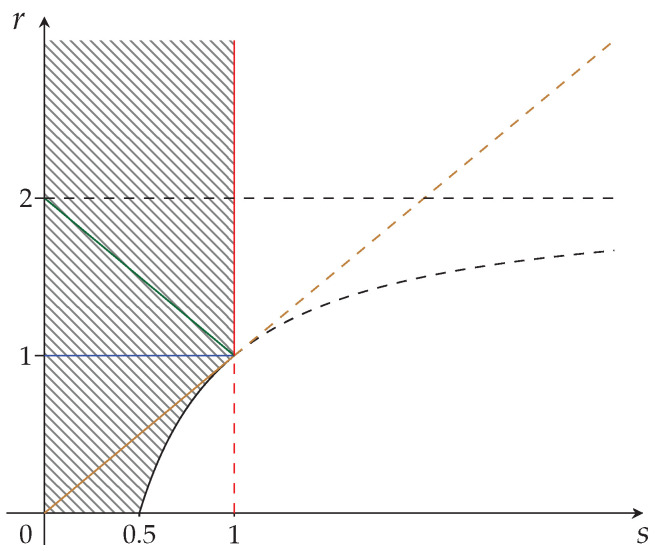
$R_{\mathrm{IV}}\cup R_{I}\cup R_{\mathrm{III}}$ is the reduction of the region of Figure 3 by taking into consideration the fully synergetic distributions only.

In order to find the reduced region corresponding to Figure 3, analogously to what has been done for Figure 1, we also need the subregions R${}_{I}$, R${}_{\mathrm{III}}$, Equations (27) and (29): the resulting subregion of fully synergetic distributions is given by $R_{\mathrm{IV}}\cup R_{I}\cup R_{\mathrm{III}}$ and is depicted in Figure 5.

## 6. Hölder Inequalities and GKS Inequalities: A Possible Conjecture

In this section, we study the relation between GKS inequalities [2] and Hölder inequalities by using examples of distributions obtained from databases of protein domain families. In order to start, some definitions and properties of the probabilistic space are now in order.

Let us first introduce the definition of the conditional probability of occurrence of the escort probability of occurrence [12]. This is a simple application to escort probabilities of Equation (3):

(44) $${\hat{p}}_{j_{1}\ldots j_{t}}\left(a_{1},\ldots ,a_{t-1}|a_{t}\right)=\frac{{\hat{p}}_{j_{1}\ldots j_{t}}\left(a_{1},\ldots ,a_{t}\right)}{{\hat{p}}_{j_{t}}\left(a_{t}\right)}\;.$$

From the definitions of escort probabilities, Equation (9), we can write

(45) $${\hat{p}}_{j_{1}\ldots j_{t}}\left(a_{1},\ldots ,a_{t}\right)=\frac{{\left(p_{j_{1}\ldots j_{t}}\left(a_{1},\ldots ,a_{t}\right)\right)}^{s}}{\sum_{b_{1},\ldots ,b_{t}}{\left(p_{j_{1}\ldots j_{t}}\left(b_{1},\ldots ,b_{t}\right)\right)}^{s}}\;,$$

and

(46) $${\hat{p}}_{j_{t}}\left(a_{t}\right)=\frac{{\left(p_{j_{t}}\left(a_{t}\right)\right)}^{s}}{\sum_{b_{t}}{\left(p_{j_{t}}\left(b_{t}\right)\right)}^{s}}\;.$$

In Equations (44)–(46), the symbols $a_{1},\ldots ,a_{t}$; $b_{1},\ldots ,b_{t}$ assume the representative letters of the one-letter code for the 20 amino acids, $a_{j}$; $b_{j}$ (1≤j≤t)∈ {A, C, D, E, F, G, H, I, K, L, M, N, P, Q, R, S, T, V, W, Y}.

After substituting Equations (45) and (46) into Equation (44), we get

(47) $${\hat{p}}_{j_{1}\ldots j_{t}}\left(a_{1},\ldots ,a_{t-1}|a_{t}\right)=\frac{{\left(p_{j_{1}\ldots j_{t}}\left(a_{1},\ldots ,a_{t-1}|a_{t}\right)\right)}^{s}{\left(p_{j_{t}}\left(a_{t}\right)\right)}^{s}}{\sum_{b_{1},\ldots ,b_{t}}{\left(p_{j_{t}}\left(b_{t}\right)\right)}^{s}{\left(p_{j_{1}\ldots j_{t}}\left(b_{1},\ldots ,b_{t-1}|b_{t}\right)\right)}^{s}\cdot {\hat{p}}_{j_{t}}\left(a_{t}\right)}\;,$$

and from Equation (46)

(48) $${\hat{p}}_{j_{1}\ldots j_{t}}\left(a_{1},\ldots ,a_{t-1}|a_{t}\right)=\frac{{\left(p_{j_{1}\ldots j_{t}}\left(a_{1},\ldots ,a_{t-1}|a_{t}\right)\right)}^{s}}{\sum_{b_{1},\ldots ,b_{t}}{\hat{p}}_{j_{t}}\left(b_{t}\right){\left(p_{j_{1}\ldots j_{t}}\left(b_{1},\ldots ,b_{t-1}|b_{t}\right)\right)}^{s}}\;.$$

We also write the definition of escort probability of occurrence of the conditional probability of occurrence [12]

(49) $$\hat{p_{j_{1}\ldots j_{t}}\left(a_{1},\ldots ,a_{t-1}|a_{t}\right)}=\frac{{\left(p_{j_{1}\ldots j_{t}}\left(a_{1},\ldots ,a_{t-1}|a_{t}\right)\right)}^{s}}{\sum_{b_{1},\ldots ,b_{t-1}}{\left(p_{j_{1}\ldots j_{t}}\left(b_{1},\ldots ,b_{t-1}|b_{t}\right)\right)}^{s}}\;.$$

We can check the definitions of Equations (48) and (49) from the equality of the two escort probabilities with the original conditional probability, for s=1

(50) $$s=1\;\Rightarrow \;{\hat{p}}_{j_{1}\ldots j_{t}}\left(a_{1},\ldots ,a_{t-1}|a_{t}\right)=\hat{p_{j_{1}\ldots j_{t}}\left(a_{1},\ldots ,a_{t-1}|a_{t}\right)}=p_{j_{1}\ldots j_{t}}\left(a_{1},\ldots ,a_{t-1}|a_{t}\right)\;.$$

We should note that the denominators of the right-hand sides of Equations (48) and (49), or,

(51) $$Z\equiv \sum_{a_{1},\ldots ,a_{t}}{\hat{p}}_{j_{t}}\left(a_{t}\right){\left[p_{j_{1}\ldots j_{t}}\left(a_{1},\ldots ,a_{t-1}|a_{t}\right)\right]}^{s}=\frac{\alpha_{j_{1}\ldots j_{t}}^{\left(s\right)}}{\alpha_{j_{t}}^{\left(s\right)}}\;,$$

and

(52) $$X\left(a_{t}\right)\equiv \sum_{a_{1},\ldots ,a_{t-1}}{\left(p_{j_{1}\ldots j_{t}}\left(a_{1},\ldots ,a_{t-1}|a_{t}\right)\right)}^{s}$$

will be equal if all amino acids in the $j_{t}$ column are equal. If we have, for instance, the $j_{t}$ column given by:

(53) $$j_{t}\;↔\;\underset{m}{\underset{︸}{(A,A,A,A,\ldots ,A)}}\;.$$

The unit vectors of probabilities ${\hat{p}}_{j_{t}}$ and $p_{j_{t}}$ will also be equal and given by

(54) $${\left({\hat{p}}_{j_{t}}\right)}^{T}={\left(p_{j_{t}}\right)}^{T}=\underset{20}{\underset{︸}{\left(1,0,0,0,\ldots ,0\right)}}\;.$$

This means that for this special case of an event of rare occurrence, we also have the equality of the conditional of the escort probability and the escort probability of the conditional probability, or the left-hand sides of Equations (48) and (49), respectively.

For a $j_{t}$-column with a generic distribution of amino acids, the denominators *Z* and $X(a_{t})$ on the right-hand sides of Equations (48) and (49) will no longer be equal. An ordering of these denominators should be decided from the probabilities of amino acid occurrence in a chosen protein domain family.

This study is undertaken with the help of the functions *Z* and $X(a_{t})$ of Equations (51) and (52) and with the functions *J* and *U*, defined below:

(55) $$J\equiv \sum_{a_{1},\ldots ,a_{t-1}}{\left[\sum_{a_{t}}{\hat{p}}_{j_{t}}\left(a_{t}\right)p_{j_{1}\ldots j_{t}}\left(a_{1},\ldots ,a_{t-1}|a_{t}\right)\right]}^{s}\;,$$

(56) $$U\equiv \sum_{a_{1},\ldots ,a_{t-1}}{\left(p_{j_{1}\ldots j_{t-1}}\left(a_{1},\ldots ,a_{t-1}\right)\right)}^{s}\equiv \alpha_{j_{1}\ldots j_{t-1}}^{\left(s\right)}\;.$$

Our method will then be the comparison of pairs of functions in order to proceed with the search for the effect of fully synergetic distributions of amino acids.

There are six comparisons to study:

(I) $X(a_{t})≷Z$

or

(57) $$\frac{1}{{\left(p_{j_{t}}\left(a_{t}\right)\right)}^{s}}\sum_{a_{1},\ldots ,a_{t-1}}{\left(p_{j_{1}\ldots j_{t}}\left(a_{1},\ldots ,a_{t}\right)\right)}^{s}≷\frac{\alpha_{j_{1}\ldots j_{t}}^{\left(s\right)}}{\alpha_{j_{t}}^{\left(s\right)}}\;;\;\;p_{j_{t}}\left(a_{t}\right)\neq 0\;.$$

(II) $X(a_{t})≷J$

or

(58) $$\begin{array}{cc} \frac{1}{{\left(p_{j_{t}}\left(a_{t}\right)\right)}^{s}}\sum_{a_{1},\ldots ,a_{t-1}}{\left(p_{j_{1}\ldots j_{t}}\left(a_{1},\ldots ,a_{t}\right)\right)}^{s}≷\frac{1}{\alpha_{j_{t}}^{\left(s\right)}}\sum_{a_{1},\ldots ,a_{t-1}}{\left[\sum_{a_{t}}{\left(p_{j_{t}}\left(a_{t}\right)\right)}^{s-1}p_{j_{1}\ldots j_{t}}\left(a_{1},\ldots ,a_{t}\right)\right]}^{s}\;; & \\ p_{j_{t}}\left(a_{t}\right)\neq 0\;. \end{array}$$

(III) $X(a_{t})≷U$

or

(59) $$\frac{1}{{\left(p_{j_{t}}\left(a_{t}\right)\right)}^{s}}\sum_{a_{1},\ldots ,a_{t-1}}{\left(p_{j_{1}\ldots j_{t}}\left(a_{1},\ldots ,a_{t}\right)\right)}^{s}≷\alpha_{j_{1}\ldots j_{t-1}}^{\left(s\right)}\;.$$

(IV) U≷J

or

(60) $$\sum_{a_{1},\ldots ,a_{t-1}}{\left(p_{j_{1}\ldots j_{t-1}}\left(a_{1},\ldots ,a_{t-1}\right)\right)}^{s}\equiv \alpha_{j_{1}\ldots j_{t-1}}^{\left(s\right)}≷\frac{H}{{\left(\alpha_{j_{t}}^{\left(s\right)}\right)}^{s}}\;,$$

where H is defined by,

(61) $$H\equiv \sum_{a_{1},\ldots ,a_{t-1}}{\left[\sum_{a_{t}}{\left(p_{j_{t}}\left(a_{t}\right)\right)}^{s-1}p_{j_{1}\ldots j_{t}}\left(a_{1},\ldots ,a_{t}\right)\right]}^{s}\;.$$

(V) J≷Z

or

(62) $$\sum_{a_{1},\ldots ,a_{t-1}}{\left[\sum_{a_{t}}{\hat{p}}_{j_{t}}\left(a_{t}\right)p_{j_{1}\ldots j_{t}}\left(a_{1},\ldots ,a_{t-1}|a_{t}\right)\right]}^{s}\equiv \frac{H}{{\left(\alpha_{j_{t}}^{\left(s\right)}\right)}^{s}}≷\frac{\alpha_{j_{1}\ldots j_{t}}^{\left(s\right)}}{\alpha_{j_{t}}^{\left(s\right)}}\;.$$

(VI) U≷Z

or

(63) $$\sum_{a_{1},\ldots ,a_{t-1}}{\left(p_{j_{1}\ldots j_{t-1}}\left(a_{1},\ldots ,a_{t-1}\right)\right)}^{s}\equiv \alpha_{j_{1}\ldots j_{t-1}}^{\left(s\right)}≷\frac{\alpha_{j_{1}\ldots j_{t}}^{\left(s\right)}}{\alpha_{j_{t}}^{\left(s\right)}}\;.$$

Equations (57)–(59) should be multiplied by ${\left(p_{j_{t}}\left(a_{t}\right)\right)}^{s}$ and after that, each one has to be summed over $a_{t}$. We then have, respectively,

(64) $$\alpha_{j_{1}\ldots j_{t}}^{\left(s\right)}≷\alpha_{j_{1}\ldots j_{t}}^{\left(s\right)}\;,$$

(65) $${\left(\alpha_{j_{t}}^{\left(s\right)}\right)}^{s-1}\alpha_{j_{1}\ldots j_{t}}^{\left(s\right)}≷H\;,$$

(66) $$\alpha_{j_{1}\ldots j_{t}}^{\left(s\right)}≷\alpha_{j_{t}}^{\left(s\right)}\cdot \alpha_{j_{1}\ldots j_{t-1}}^{\left(s\right)}\;.$$

Equations (60), (62) and (63) can be written, respectively, as

(67) $${\left(\alpha_{j_{t}}^{\left(s\right)}\right)}^{s}\cdot \alpha_{j_{1}\ldots j_{t-1}}^{\left(s\right)}≷H$$

(68) $$H≷{\left(\alpha_{j_{t}}^{\left(s\right)}\right)}^{s-1}\cdot \alpha_{j_{1}\ldots j_{t}}^{\left(s\right)}\;,$$

(69) $$\alpha_{j_{t}}^{\left(s\right)}\cdot \alpha_{j_{1}\ldots j_{t-1}}^{\left(s\right)}≷\alpha_{j_{1}\ldots j_{t}}^{\left(s\right)}\;,$$

The Hölder’s inequality as applied to probabilities of occurrence [3] is written as

(70) $$\frac{1}{{\left(\alpha_{j_{t}}^{\left(s\right)}\right)}^{s}}{\left[\sum_{a_{t}}{\left(p_{j_{t}}\left(a_{t}\right)\right)}^{s-1}p_{j_{1}\ldots j_{t}}\left(a_{1},\ldots ,a_{t}\right)\right]}^{s}\geq \frac{\sum_{a_{t}}{\left[p_{j_{1}\ldots j_{t}}\left(a_{1},\ldots ,a_{t}\right)\right]}^{s}}{\alpha_{j_{t}}^{\left(s\right)}}\;.$$

After multiplying by ${\left(\alpha_{j_{t}}^{\left(s\right)}\right)}^{s}$ and summing over $a_{1},\ldots ,a_{t-1}$, we get

(71) $$H\geq {\left(\alpha_{j_{t}}^{\left(s\right)}\right)}^{s-1}\alpha_{j_{1}\ldots j_{t}}^{\left(s\right)}\;\;,\;s\leq 1\;.$$

We also define

(72) $$O\equiv {\left(\alpha_{j_{t}}^{\left(s\right)}\right)}^{s-1}\alpha_{j_{1}\ldots j_{t}}^{\left(s\right)}\;,$$

(73) $$B\equiv {\left(\alpha_{j_{t}}^{\left(s\right)}\right)}^{s}\alpha_{j_{1}\ldots j_{t-1}}^{\left(s\right)}\;.$$

We then summarize the results obtained:

1. Equation (64) is only an identity: $\alpha_{j_{1}\ldots j_{t}}^{\left(s\right)}=\alpha_{j_{1}\ldots j_{t}}^{\left(s\right)}$.
2. Equations (65) and (68) can be ordered by Hölder’s inequality, Equations (70) and (71).
3. Equations (66) and (69) can be ordered by GKS inequalities, corresponding to fully synergetic distributions of amino acids, $\alpha_{j_{1}\ldots j_{t}}^{\left(s<1\right)}\leq \alpha_{j_{t}}^{\left(s<1\right)}\cdot \alpha_{j_{1}\ldots j_{t-1}}^{\left(s<1\right)}$.
4. Equation (67) cannot be ordered without additional experimental/phenomenological information on the probabilities of occurrence to be obtained from updated versions of protein domain family databases [13].

We now collect the formulae obtained from the analysis performed on this section. Equations (65) and (68) are ordered by Hölder’s inequality. We write

(74) H−O≥0,s<1.

Equations (66) and (69) are ordered by GKS inequality. We write

(75) B−O≥0,s<1.

After using Equation (73), we can write Equation (67) as

(76) B−H≷0.

**Figure 6:**
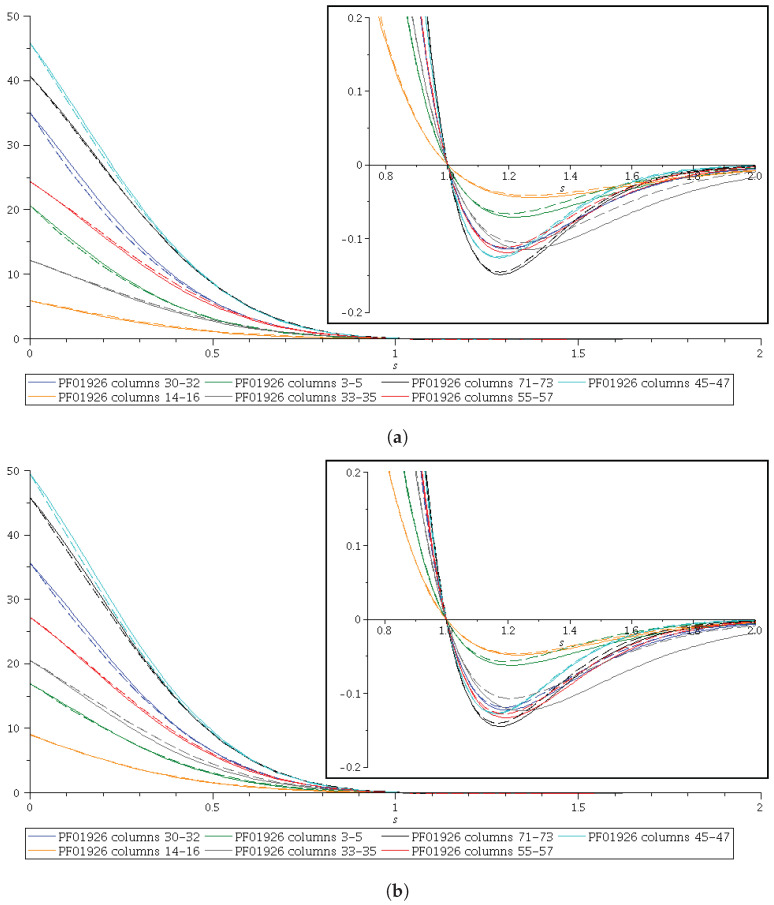
Hölder (H−O≥0, 0≤s≤1) distributions (dashed curves) and Khinchin–Shannon (B−O≥0, 0≤s≤1) distributions (continuous curves) of the PF01926 protein domain family from (**a**) protein domain family PF01926 obtained from Pfam 27.0 and (**b**) protein domain family PF01926 obtained from Pfam 35.0. The top-right inset shows details of the curves for s≥1.

In Figure 6a,b we have depicted the curves corresponding to functions H−O and B−O for seven 3-sets of contiguous columns and 80 rows, chosen from databases Pfam 27.0 and Pfam 35.0, respectively. There are also inset figures in order to show the curves for s≥1.

**Figure 7:**
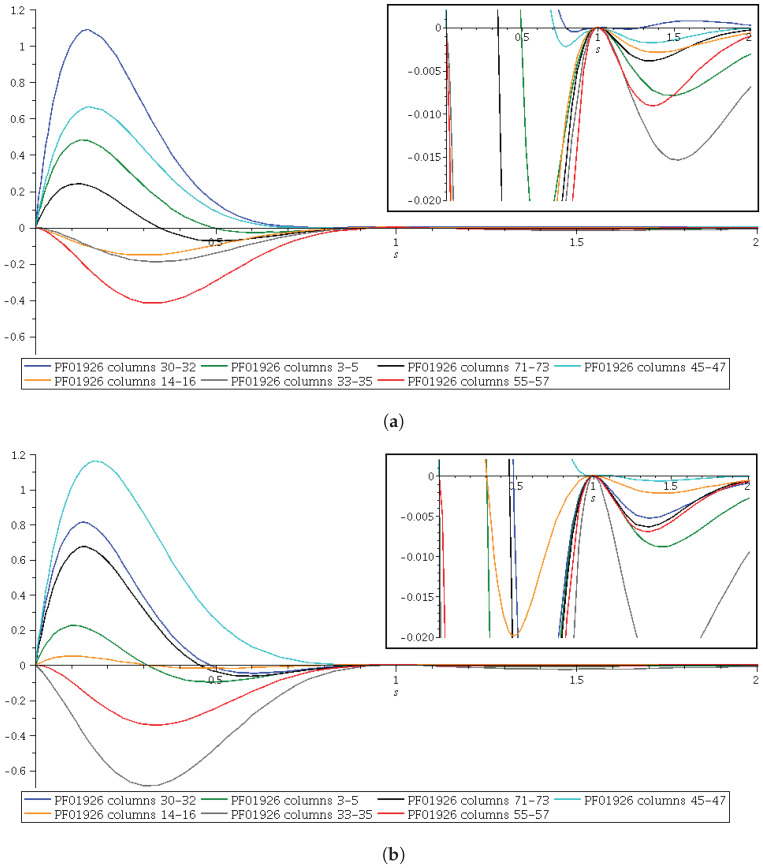
B−H difference of the PF01926 protein domain family from (**a**) protein domain family PF01926 obtained from Pfam 27.0 and (**b**) protein domain family PF01926 obtained from Pfam 35.0. The top-right inset shows details of the curves for s≥1. B−H≥0, H−O≥0 ⟹ B−O≥0.

In Figure 7a,b, we do the same for the differences B−H. We emphasize that for the 3-sets such that B−H≥0, 0≤s≤1, the GKS inequalities B−O≥0 will result from the validity of Hölder’s inequality. We have worked with the PF01926 protein domain family to perform all the calculations.

## 7. Concluding Remarks

The first comment we want to make to the present work is about the possibility of working in a region of the parameter space that preserves the strict concavity and the fully synergetic structure of the Sharma–Mittal class of entropy measure distributions to be visited by solutions of a new successful statistical mechanics approach. The usual work with Havrda–Charvat distributions describes the evolution along the boundary (r=s) of the region (r≥s>0) that was correctly considered to correspond to strict concavity, but it is also known to be non-synergetic for s>1. We now have the opportunity to develop this statistical mechanics approach along an extended boundary, preserving the strict concavity and providing the study of the evolution of fully synergetic entropy distributions. A first sketch of these developments will be presented in a forthcoming publication.

With respect to Figure 6 and Figure 7, we could hypothesize that if the ordering of *B* and H could not be obtained, this would be due to the poor alignment of some protein domain families we have been using, but we are not confident enough that we could do this, because we would need much more information “in silico” to be obtained from many other protein domain families. In other words, we expect that a good alignment of a protein domain family will result in the ordering of *B* and H, but we need to verify this in a large number of families from different Pfam versions before we proceed with a proposal of a method to improve the Pfam database. This looks promising for good scientific work in the line of research we have been aiming to introduce in Ref. [2] and in this contribution.

## Data Availability

Not applicable.

## Abbreviations

The following abbreviation is used in this manuscript:

GKS	Generalized Khinchin-Shannon
